# Nanoparticle capture of urinary lipoarabinomannan for diagnosing childhood tuberculosis

**DOI:** 10.1128/jcm.01559-25

**Published:** 2026-10-12

**Authors:** Kumail Ahmed, Brendan T. Mullen, Najeeha T. Iqbal, Tania A. Thomas

**Affiliations:** 1 Department of Paediatrics and Child Health, Aga Khan University 196391, Karachi, Pakistan; 2 Department of Internal Medicine, University of Virginia 2358, Charlottesville, Virginia, USA; 3 Department of Medicine, Division of Allergy and Infectious Diseases, University of Washington, Seattle, Washington, USA; 4 Division of Infectious Diseases and International Health, University of Virginia 2358, Charlottesville, Virginia, USA; University of Western Australia, Perth, Australia

**Keywords:** diagnostics, pediatric tuberculosis, tuberculosis

## Abstract

Urinary assays detecting lipoarabinomannan (LAM) as a diagnostic test for tuberculosis (TB) have limited sensitivity in pediatric populations. We aimed to evaluate the Ceres TB-Nanotrap to concentrate LAM and augment detection in urine using the Alere lateral flow assay (LF-LAM). This case-control study recruited 32 children with and without TB aged 1–18 years in Karachi, Pakistan, with banked urine specimens. TB case participants were classified as confirmed or unconfirmed TB using modified National Institutes of Health (NIH) consensus criteria. The LF-LAM test was performed before and after concentrating 5 mL of urine with TB-Nanotrap particles. Band intensity was measured via visual grading and digital quantification. Urine LAM sensitivity by visual grading was 4.5% (95% confidence interval [CI] 0.3–18.5) pre-concentration and 50.0% (95% CI 30.0–70.0) post-concentration. Sensitivity by digital quantification was 0.0% (95% CI 0.0–15.4) pre-concentration and 63.9% (95% CI 42.8–81.4) post-concentration. Specificity was high with both methods (visual grading: 90.0% [95% CI 62.8–99.4] pre-concentration and 90.0% [95% CI 62.8–99.4] post-concentration; digital analysis: 80.0% [95% CI 44.4–97.5] and 90.0% [95% CI 62.8–99.4], respectively). For cases, digital analysis showed an increase in median LF-LAM band intensity from 0.0 arbitrary units (AU) (interquartile range [IQR] 0.00–32.5) in unconcentrated samples to 98.4 AU (IQR 34.9–212.2) in concentrated samples. Concentration with TB-Nanotrap greatly increased sensitivity of urine LAM detection without a change in specificity. Use of TB-Nanotrap and digital quantitative analysis of urine LAM improved the diagnostic accuracy of the LF-LAM assay in this pediatric population and should be validated in larger studies.

**IMPORTANCE:**

Diagnosing TB in children is challenging due to a lack of accurate and accessible diagnostic tests. In this study, we evaluated two methods of improving detection of TB in children using a commercially available urine test. First, we processed the urine to concentrate any available TB antigens. Second, we included a digital reader to interpret the test results. We found that concentrating urine and using a digital reader led to an increase in TB detection among children who had been diagnosed with TB, while minimizing false-positive results among children who did not have TB.

## INTRODUCTION

To date, an accurate test to confirm a diagnosis of tuberculosis (TB) in children does not exist. The most common method of microbiologically confirming TB relies on detecting mycobacteria from respiratory specimens using molecular methods, microscopy, or culture. However, reliance on respiratory specimens does not meet the needs of most children due to logistic and physiological limitations in collecting a high-quality sample reliably, particularly since childhood TB often represents a paucibacillary disease state. As such, there is a pressing need for novel, pediatric-friendly methods to diagnose TB (1, 2). Evaluating TB biomarkers from urine holds numerous advantages, including the potential to collect large volumes through minimally invasive means (3, 4). Additionally, a urinary biomarker may obviate the need to localize the exact site of infection, which is especially useful in childhood TB when symptoms may be non-specific and extrapulmonary manifestations are common.

Diagnostic development efforts have focused on detecting lipoarabinomannan (LAM), an outer cell wall mycobacterial glycolipid antigen, from urine specimens among people with active TB disease (5). The Alere Determine TB LAM lateral flow assay (LF-LAM), the only WHO-endorsed point-of-care test for TB, has limited sensitivity in populations with TB without HIV co-infection (6), including children (7, 8). The LF-LAM assay performs best in individuals with advanced HIV (CD4+ T cells ≤100) with a sensitivity of 54% and a specificity of 88% (9). In addition to concerns about reliability outside of this population, visual assay interpretation of the LF-LAM, the need for CD4 cell count results, and narrow target population have been cited by users as limitations to uptake (10).

Efforts to optimize this assay for expansion into HIV-negative populations include incorporating a urine processing step to concentrate LAM antigens using nanoparticle-based technology to augment detection through commercially available assays. The Nanotrap (Ceres Nanosciences, Manassas, VA, USA) employs hydrogel acrylamide microparticles with chemical affinity dyes, which attract target analytes in solution. Specimen processing using this technology can concentrate low-abundant molecules and remove proteins or other interfering substances, thereby optimizing the performance of currently available testing platforms. This technology has been applied successfully to infectious diseases such as Lyme, toxoplasmosis, emerging viral pathogens, and wastewater analysis (11–13). A TB-Nanotrap has also been created using Reactive Blue 221 as the high-affinity dye that couples well with the 5-methylthio-d-xylofuranose epitope of LAM. Initial studies to detect urinary LAM with TB-Nanotraps and the LF-LAM assay among HIV-negative adults with active TB yielded a sensitivity of 96% and a specificity of 81% (14). However, validation studies in pediatric populations are lacking. Here, we describe a proof-of-concept study evaluating the Ceres TB-Nanotrap as a method of augmenting detection of LAM antigens in urine among children with and without TB using the LF-LAM assay. We also aimed to assess agreement between readers of assay results and the impact of digital LAM assay band quantification on assay performance.

## MATERIALS AND METHODS

### Study design and population

This work incorporates a case-control design in a 2:1 ratio among children who were enrolled in a parent study examining a blood-based immunodiagnostic assay. The parent studies recruited children 1–18 years of age from outpatient clinics in the greater Karachi area of Pakistan (15). Physicians at these clinics referred children based on clinical or microbiological diagnoses of pulmonary or extrapulmonary TB (“case”). After parental permission and assent were obtained, participants underwent evaluation with standardized data collection, including clinical and radiographic information. A tuberculin skin test was performed and interpreted according to local guidelines. Chest radiography was obtained. Specimen collection included blood and urine samples from all and sputum collection via expected sputum for children >8 years. Gastric aspirates were considered when clinically feasible for younger children per local guidelines. Participants returned after 4–6 weeks for repeat clinical evaluation.

Asymptomatic children (“controls”) were recruited from the same settings and enrolled if they had no history of TB disease or TB exposure, no current clinical suspicion of TB, and no history of major illnesses in the last 2 weeks. After parental permission and assent were obtained, participants underwent evaluation with standardized data collection. Asymptomatic participants returned after 4–6 weeks to ensure clinical stability. Participants (cases and controls) were ineligible for the parent study if they had received BCG vaccine or tuberculin skin test within the past 8 weeks. Full inclusion and exclusion criteria are described in File S1. All case and control participants were reviewed, and a random sampling of case and control participants with sufficient quantities of stored urine was selected for inclusion in the study. The total sample size was limited by reagent availability. Updated National Institutes of Health (NIH) consensus criteria were used to further categorize these participants as “confirmed” or “unconfirmed” TB and “non-TB controls” (16). Per these definitions, confirmed cases had at least one sample positive by GeneXpert. Unconfirmed cases had clinical features suggestive of TB and at least two supportive findings between radiographic findings of TB, immunological evidence of TB exposure, or clinical response to TB treatment.

### Urine sample collection and processing

All participants provided a spot urine sample, collected non-invasively via clean catch, which was divided into two 5 mL aliquots and stored at −80°C for future batch analyses. Urine samples were stored from 6 months to 5 years, depending on the time of enrollment. Urine specimens were thawed and centrifuged at 10,000 × *g* for 5 min at room temperature per standardized operating procedures, as developed by the Centers for Disease Control and Prevention (CDC) and ASM through the Mentor4TB Program (17). A baseline LF-LAM test was performed using 60 µL of the supernatant and interpreted after 25 min with use of the reference scale card according to package instructions (18). The remaining 5 mL of the supernatant was processed with the Ceres magnetic TB-Nanotrap particles. Briefly, 400 µL of TB-Nanotrap particles was placed in a magnetic rack to remove the storage buffer solution. TB-Nanotrap particles were subsequently resuspended in 125 µL of 2% SDS solution before adding the urine, followed by incubation for 30 min on an orbital shaker at room temperature. Using a magnetic rack to attract the TB-Nanotrap particles, we discarded the supernatant. Elution buffer (75 µL of 2% Tween 20 solution) was added and incubated for 15 min on an orbital shaker to free LAM from nanocages. Finally, using a magnetic rack to separate TB-Nanotrap particles from the eluted sample, we removed 60 µL of the processed sample, loaded it on the LF-LAM test strip, and interpreted it. LF-LAM tests were initially visually interpreted in real time in a non-blinded fashion (KA, reviewer 1); photographs of the test strips were captured using a camera and lightbox with a standardized light source. An independent reviewer blinded to TB classification and assay concentration status performed a second interpretation of light-standardized photographs of all test results (TAT, reviewer 2); any discrepant results were adjudicated by a third, independent, blinded reviewer (BTM, reviewer 3) via interpretation of photographs. All results were recorded qualitatively (positive/negative), as well as quantitatively on a 4-point scale according to band intensity. As per manufacturer instructions, a positive result required the appearance of a band in the “test” window of at least grade 1 intensity as well as in the “control” window. Tests were deemed negative if there was no visible band in the test window, but the control window showed a visible band.

### Digital image analysis

To quantify band intensity in each sample before and after concentration with the TB-Nanotrap, images of each lateral flow assay test strip were photographed using a lightbox with standardized light source, distance, and lighting conditions. Photos were then imported to ImageJ (National Institutes of Health, Bethesda, MA, USA) for analysis. For each assay image, the peak intensities of the control band and result band were measured. Band intensity was measured in mean arbitrary units (AU). For the purposes of determining performance characteristics, a positive assay was considered one in which the result band had equal or higher intensity than the reference card grade-1 band. Quantitative band intensity was measured as AU relative to the reference card grade-1 band; values less than the reference card band were reported as zero.

### Statistical analysis

Simple frequencies and measures of central tendency were used to report demographic and clinical data. To explore potential differences in diagnostic accuracy with the TB-Nanotrap, performance measures, including sensitivity and specificity, were calculated as percentages with 95% confidence intervals (CIs) using confirmed and unconfirmed TB as a composite reference standard to calculate sensitivity. We compared performance measures based on results of visual grading between TB-Nanotrap concentrated and unconcentrated urine. We also compared test performance based on results of digital analysis between TB-Nanotrap concentrated and unconcentrated urine. To assess for reviewer agreement and potential bias of LAM interpretation related to the blinding status of reviewer 1, Cohen’s kappa statistics were performed for different reviewer pairs. Data analysis was performed with SPSS 28.0 (IBM, Armonk, NY).

## RESULTS

### Participant demographics

A total of 167 case participants, confirmed and unconfirmed, were reviewed for potential inclusion in the study. One hundred fourteen case participants had available urine specimens of at least 5 mL volume. A total of 49 control participants were reviewed for potential inclusion, and all had available urine specimens. We included a total of 32 randomly sampled, eligible case and control participants, recruited from April to July 2015 and from September 2018 to September 2020, with demographic and clinical data displayed in Table 1. There were 22 participants meeting criteria for the “TB case” definition, which included 10 (31.3%) participants with GeneXpert MTB/RIF confirmed TB and 12 (37.5%) participants who were diagnosed based on clinical criteria. Of the 10 participants who were GeneXpert MTB/RIF confirmed, 8 had pulmonary TB. Two had extrapulmonary TB presenting as central nervous system disease with cerebrospinal fluid samples positive by GeneXpert MTB/RIF. Ten participants were included in the control group. Cases (median age 7.5 years, interquartile range [IQR] 5.0–14.0) were younger than controls (14.0 years, IQR 5.0–18.0).

**TABLE 1:** Demographic and clinical characteristics

Characteristic	Overall (*n* = 32)	Cases (*n* = 22)		Controls (*n* = 10)
		Unconfirmed (***n*** = 12)	Confirmed (***n*** = 10)	
Age distribution, *n* (%)				
2–5 years	9 (28)	4 (33.3)	2 (20.0)	3 (30)
6–9 years	7 (22)	6 (50.0)	0 (0.0)	1 (10)
10–14 years	7 (22)	2 (16.7)	0 (0.0)	2 (20)
15–18 years	9 (28)	0 (0.0)	8 (80.0)	4 (40)
Female, *n* (%)	17 (53)	4 (33.3)	7 (70.0)	6 (60)
Cough present, *n* (%)		9 (75.0)	8 (80.0)	
Any imaging finding of TB present on chest X-ray, *n* (%)^*a*^		12 (100.0)	8 (80.0)	
Extrapulmonary TB, *n* (%)^*b*^		0 (0)	2 (20)	

^*a*^ Chest X-ray findings: lymphadenopathy, opacification, miliary deposits, cavities, and calcifications. Available for 21/22 cases.

^*b*^ *n* (%) within each case classification with extrapulmonary TB.

### LAM assay visual grading

**Fig 1:**
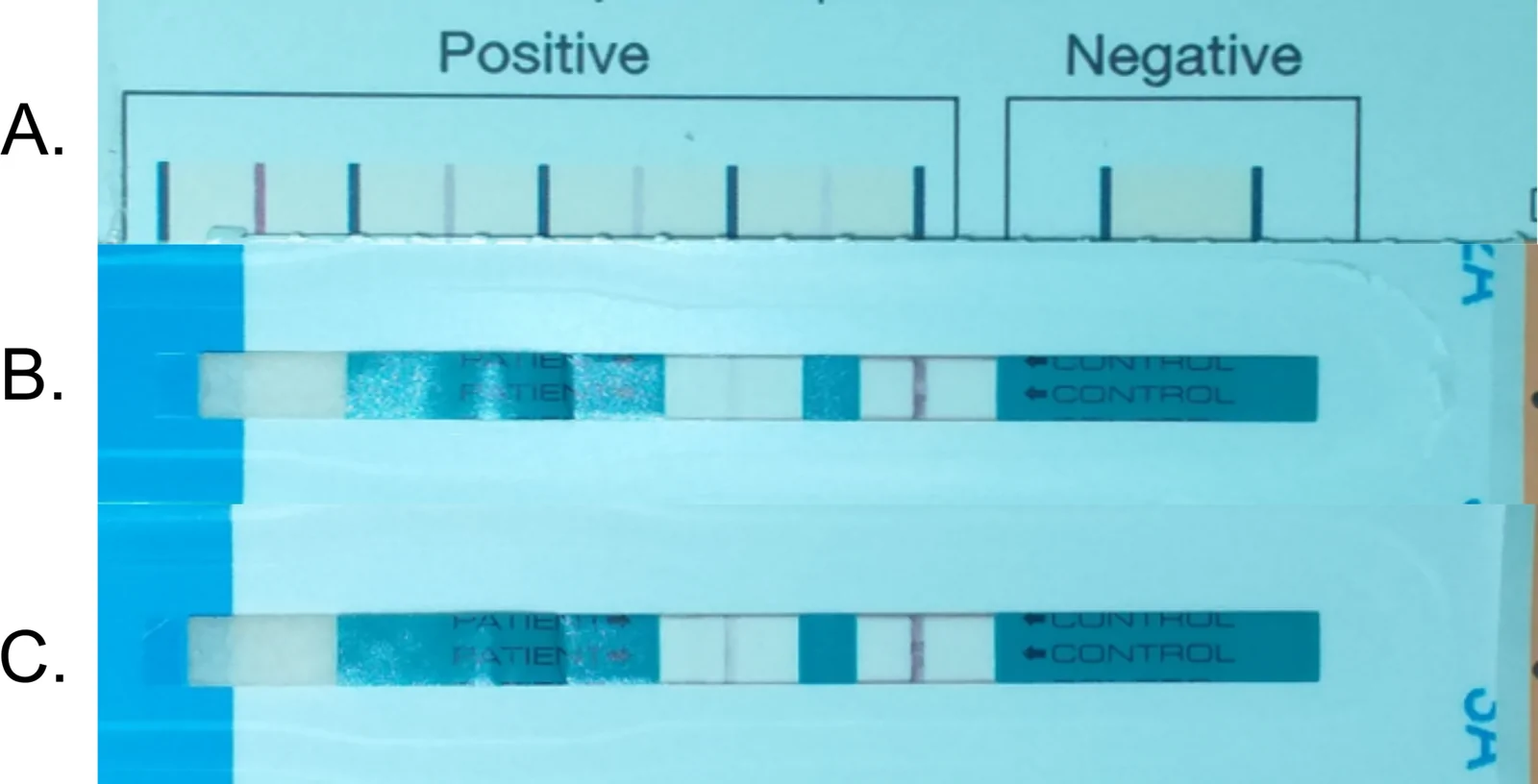
LF-LAM (**A**) reference card, (**B**) assay before TB-Nanotrap concentration, and (**C)** assay after TB-Nanotrap concentration in a participant with unconfirmed TB.

Figure 1 gives an example of the differences in LF-LAM assay results in urine from a single participant with unconfirmed TB before and after TB-Nanotrap concentration. The LF-LAM assay was read as negative by reviewer 1 and reviewer 2 before concentration and positive by reviewer 1 and reviewer 2 after concentration.

**Fig 2:**
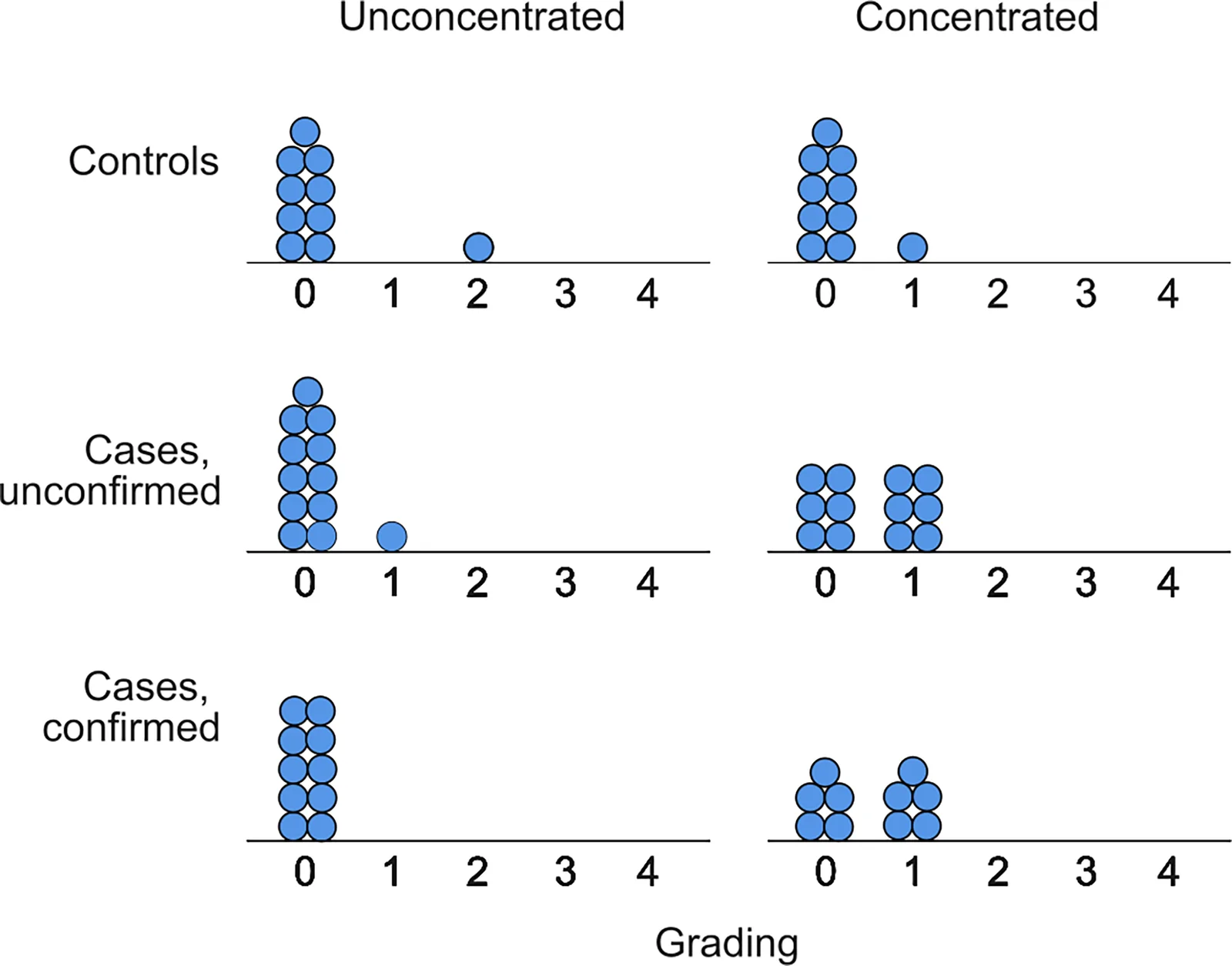
Quantitative changes in band intensity by consensus visual grading after concentration.

Fig. 2 shows counts of LF-LAM assays by grade for both controls and cases. There were 9 out of 10 control participants who had a negative LF-LAM result before and after TB-Nanotrap concentration. However, one teenage control participant had a positive LF-LAM result that demonstrated a grade 2 band intensity before TB-Nanotrap concentration and grade 1 band intensity after TB-Nanotrap concentration. Among case participants, only 1 out of 22 had a positive LF-LAM assay with a grade of 1 prior to TB-Nanotrap concentration. After concentration, 11 of 22 had a positive LF-LAM assay, all with a grade of 1. Ages did not differ between cases with positive and negative assays after concentration (*P* = 0.41). Interobserver agreement was fair for unconcentrated assay positivity (*κ* = 0.398) and substantial for concentrated assay positivity (*κ* = 0.811). Interobserver agreements were similar between reader 3 and reader 2 (*κ* = 0.385) and between reader 3 and reader 1 (*κ* = 0.478). No invalid tests were observed.

### Diagnostic accuracy of urine LAM

The diagnostic accuracy of LF-LAM compared to the composite reference standard is shown in Table 2. When compared to the composite reference standard, visual grading of LF-LAM had a sensitivity of 4.5% (95% CI 0.3–18.5) among cases and a specificity of 90.0% (95% CI 62.8–99.4) among controls. After TB-Nanotrap concentration, sensitivity with visual grading increased to 50.0% (95% CI 30.0–70.0) with no change in specificity (Table 2). Digital analysis resulted in a further increase in sensitivity of LF-LAM after TB-Nanotrap concentration (sensitivity = 63.9% [95% CI 42.8–81.4]) (Table 2). Specificity of the assay with digital analysis was similar before and after concentration (specificity, unconcentrated = 80.0% [95% CI 44.4–97.5]; specificity, concentrated = 90.0% [95% CI 62.8–99.4]). Agreement was moderate for assay positivity between visual grading and digital analysis (*κ* = 0.585).

**TABLE 2:** Diagnostic accuracy of unprocessed and TB-Nanotrap concentrated urine LAM by visual and digital analysis

Analysis method and case grouping	% Sensitivity (95% CI)	% Specificity (95% CI)
Visual analysis		
	Unconfirmed and confirmed	Controls
LAM without concentration	4.5 (0.3–18.5)	90.0 (62.8–99.4)
LAM with concentration	50.0 (30.0–70.0)	90.0 (62.8–99.4)
	Unconfirmed	Controls
LAM without concentration	8.3 (0.5-31.9)	90.0 (62.8-99.4)
LAM with concentration	50.0 (23.8-76.2)	90.0 (62.8-99.4)
	Confirmed	Controls
LAM without concentration	0.0 (0.0–30.9)	90.0 (55.5–99.8)
LAM with concentration	50.0 (18.7–81.2)	90.0 (55.5–99.8)
Digital analysis		
	Unconfirmed and confirmed	Controls
LAM without concentration	0.0 (0.0–15.4)	80.0 (44.4–97.5)
LAM with concentration	63.9 (42.8–81.4)	90.0 (62.8–99.4)
	Unconfirmed	Controls
LAM without concentration	0.0 (0.0–26.5)	80.0 (44.4–97.5)
LAM with concentration	66.7 (34.9–90.1)	90.0 (55.5–99.8)
	Confirmed	Controls
LAM without concentration	10.0 (0.3–44.5)	80.0 (44.4–97.5)
LAM with concentration	60.0 (26.2–87.8)	90.0 (55.5–99.8)

**Fig 3:**
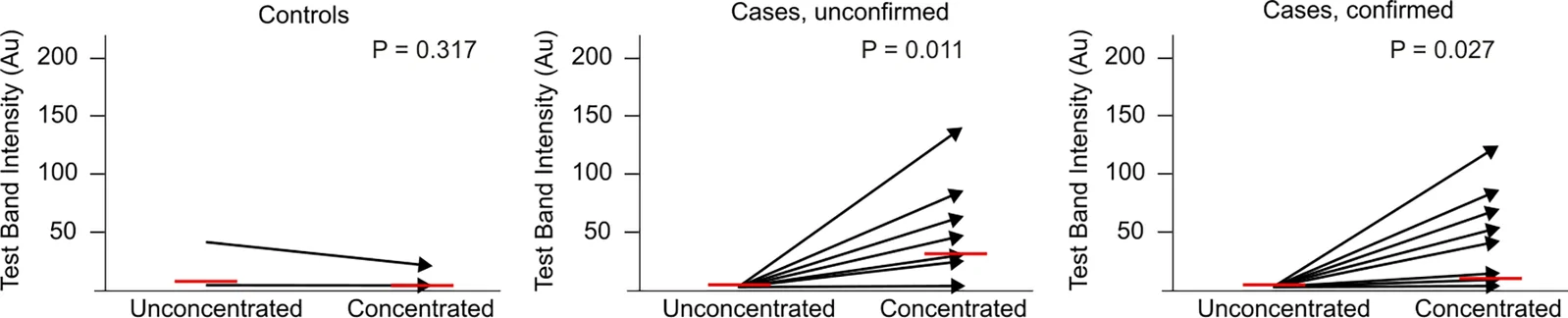
Paired test band intensity values by digital analysis before and after TB-Nanotrap concentration for each control and case. The red line indicates the median value.

Figure 3 shows the distribution of assay test band intensity values relative to reference card grade 1 bands before and after TB-Nanotrap concentration for controls, unconfirmed cases, and confirmed cases. In control participants, median band intensity did not change with concentration (unconcentrated, 0 AU, IQR 0–0; concentrated 0, IQR 0–0; *P* = 0.317). In unconfirmed cases, median band intensity increased from 0.0 (0.0–0.0) to 4.5 (0–57.5) (*P* = 0.011). In confirmed cases, there was a greater increase in band intensity from 0.0 (0.0–0.0) to 24.0 (0.0–93.3) (*P* = 0.027).

## DISCUSSION

In this cohort analysis of pediatric participants with and without TB, we characterized the diagnostic accuracy of the LF-LAM assay after processing urine samples with the TB-Nanotraps as a means of concentrating any available LAM antigen within the specimen. We found that the TB-Nanotrap increased the overall sensitivity of the urine LF-LAM assay to 50%, which approaches LF-LAM sensitivity in patients with advanced HIV (9). Importantly, concentration increased the sensitivity of LF-LAM from 8.3 to 50.0 in participants with unconfirmed TB who otherwise did not have a confirmatory test. Specificity was overall unchanged by the TB-Nanotrap processing step. TB-Nanotrap concentration may have also resulted in improved consistency of visual assay interpretation (*κ* = 0.398, unconcentrated; *κ* = 0.811, concentrated).

The WHO target product profiles for TB diagnostics (19) indicate that a non-sputum, low complexity assay should attain a sensitivity of 80%. The use of TB-Nanotrap as presented here does not meet this sensitivity target but approaches LF-LAM sensitivity in patients with advanced HIV and thus warrants further validation. This study addresses limitations that exist in currently available urine LAM tests, as outlined in the 2019 WHO updates on the use of LAM in the diagnosis of TB in people living with HIV (20) and in a more recent qualitative survey about the use of LAM in TB-endemic regions (10). Although LF-LAM has a rapid turnaround time and is available as a point-of-care test, the subjective visual assay interpretation, the need for CD4 cell count results, and the narrow target population of children and adults with advanced HIV have been identified as limitations to more widespread uptake. Here, we aimed to address several of these limitations by validating a method of urine LAM concentration with the goal of improving diagnostic accuracy in a pediatric population without HIV. Diagnosis of TB in pediatric populations with presumptive TB remains challenging. TB-Nanotrap worked equally well among children with confirmed TB and those with unconfirmed TB. TB-Nanotrap offers a significant additive diagnostic yield, with 50% of clinically diagnosed TB cases having a positive urine LAM after TB-Nanotrap. Our findings suggest that incorporating this processing step to capture urine LAM may be a useful method to increase access to rapid-diagnostic tests for TB in children who otherwise would not have laboratory confirmation of a diagnosis. This is particularly true for children who have negative TB test results by other rapid-diagnostic methods, such as the Xpert MTB/RIF. The WHO target product profile report for TB diagnostics identified that a rapid, non-sputum-based diagnostic test capable of detecting child TB is of high priority (19). Urine LAM represents a vital tool for addressing this goal, in that it is rapid and does not require sputum collection. The improved test characteristics shown here with the use of nanoparticle concentration warrant further validation in larger cohort studies of patients with and without HIV. Use of digital quantitative analysis over visual grading further increased TB-Nanotrap concentrated assay sensitivity from 50.0% to 63.9%. This quantitative increase in LF-LAM intensity in case participant samples appeared to be more pronounced in confirmed (0.0–24.0 AU) as compared to unconfirmed (0.0–4.5 AU). Further validation is needed to determine if age, bacillary burden, or other clinical factors affect assay performance. Our findings of improved test sensitivity with the use of digital interpretation of LAM band intensity lend evidence to ongoing developments to implement digital readers as part of urine LAM interpretation (21). Attempts at utilizing smartphones as an accessible, point-of-care technology for quantifying assay results have been successful in other lateral flow assays (22, 23) and could reasonably be applied to urine LAM as well.

Attempts to quantify the inter-reader variability of LAM assay interpretation have yielded heterogeneous results. In 2014, the manufacturer of LF-LAM changed the assay from a 5-point scale to a 4-point scale to assist with discriminating between a positive and negative assay. However, even after this change, studies designed to quantify this variability found significant differences in interpretation of weak positive bands in LF-LAM (24, 25). A meta-analysis of LAM assay performance called for consideration of solutions to improve assay interpretation by point-of-care users (26). Use of objective, quantitative digital grading may be one method of resolving this variability. Several electrochemiluminescence LAM assays are under ongoing investigation to potentially improve accuracy and precision (27). However, protocols for these assays typically require prolonged incubation steps as well as the use of a specialized instrument (28). The Fujifilm SILVAMP-II LAM assay is a newer LAM assay that may improve assay sensitivity, though lot-to-lot variability of the first generation of this assay has limited broader adoption (29). Methods of processing LAM, such as the use of TB-Nanotrap as presented here, may also have potential in maintaining LAM as an accessible test in resource-limited settings.

Several limitations exist to the data presented here. Analyses were performed on a small cohort of children who were not age-matched. Additionally, the use of a case-control study design introduces potential bias to accuracy estimates. During assay interpretation, reviewer 1 was not blinded to assay concentration status, which may have biased assay interpretation, although the two fully blinded reviewers had a similar level of agreement between each other and as compared to reviewer 1. Although this study enrolled a small and heterogeneous group of participants, these serve to address an important gap in data on TB diagnostics in a priority population for the development of non-sputum-based diagnostics. We used a composite clinical reference standard rather than a purely microbiological reference standard, which may have impacted the performance characteristics by including participants with more paucibacillary disease. However, this composite reference standard more accurately reflects the high proportion of children who are diagnosed with TB without microbiological confirmation (16) and adheres to consensus guidelines for studies on TB diagnostics. The inclusion of asymptomatic children as a control group, as opposed to symptomatic children adjudicated as unlikely to have TB per consensus criteria, is a potential source of bias. Further validation of this platform should include a larger cohort inclusive of symptomatic participants meeting the full spectrum of TB consensus classifications. As it stands, the assay employed here requires laboratory space, basic equipment, personnel, and time that would require further simplification for adaptation to point-of-care testing. The use of frozen urine specimens stored for 6 months to 5 years for analyses may have impacted assay performance in this study. Data on the use of frozen urine specimens in LAM assays are limited, with differing findings on LAM stability depending on freezing method and assay (30, 31). To inform future evaluations of this and other urine-based assays, LAM stability should be further validated by comparing freshly collected and stored frozen specimens across different assays and durations of storage. TB-Nanotrap concentration here did not significantly improve the specificity of the LAM assay. The inclusion of a limited number of control participants, with an older average age than case participants, may have impacted specificity. Several explanations have been proposed for limitations to specificity for urine LAM. Whether through disseminated infection or contamination, non-tuberculous mycobacteria have been shown to cross-react with urinary LAM assays (32). Efforts are underway exploring other antibody combinations to target MTB urine LAM more specifically (27).

Overall, data presented here on diagnostic accuracy of urine LAM with nanoparticle concentration and digital analysis demonstrate improved accuracy in a pediatric population. In addition to further validation studies in larger pediatric cohorts, future studies should be done to evaluate the performance of urine LAM with nanoparticle concentration in broader patient populations with limited TB diagnostic tools, and the impact of its use on clinical outcomes.

## SUPPLEMENTAL MATERIAL

File S1 jcm.01559-25-s0001.docx Inclusion/exclusion criteria and case definitions.

ASM does not own the copyrights to Supplemental Material that may be linked to, or accessed through, an article. The authors have granted ASM a non-exclusive, world-wide license to publish the Supplemental Material files. Please contact the corresponding author directly for reuse.

## References

[1] World Health Organization. 2018. Global tuberculosis report. World Health Organization, Geneva, Switzerland.

[2] World Health Organization. 2013. Roadmap for childhood tuberculosis: towards zero deaths. World Health Organization, Geneva, Switzerland.

[3] Kaufman J, Fitzpatrick P, Tosif S, Hopper SM, Donath SM, Bryant PA, Babl FE. 2017. Faster clean catch urine collection (Quick-Wee method) from infants: randomised controlled trial. BMJ 357:j1341. doi:10.1136/bmj.j1341 PMC6284210 28389435

[4] Wallis RS, Pai M, Menzies D, Doherty TM, Walzl G, Perkins MD, Zumla A. 2010. Biomarkers and diagnostics for tuberculosis: progress, needs, and translation into practice. Lancet 375:1920–1937. doi:10.1016/S0140-6736(10)60359-5 20488517

[5] Hamasur B, Bruchfeld J, Haile M, Pawlowski A, Bjorvatn B, Källenius G, Svenson SB. 2001. Rapid diagnosis of tuberculosis by detection of mycobacterial lipoarabinomannan in urine. J Microbiol Methods 45:41–52. doi:10.1016/s0167-7012(01)00239-1 11295196

[6] World Health Organization. 2015. The use of lateral flow urine lipoarabinomannan assay (LF-LAM) for the diagnosis and screening of active tuberculosis in people living with HIV: policy guidance World Health Organization, Geneva, Switzerland.

[7] Blok N, Visser DH, Solomons R, Van Elsland SL, den Hertog AL, van Furth AM. 2014. Lipoarabinomannan enzyme-linked immunosorbent assay for early diagnosis of childhood tuberculous meningitis. Int J Tuberc Lung Dis 18:205–210. doi:10.5588/ijtld.13.0526 24429314

[8] Kroidl I, Clowes P, Reither K, Mtafya B, Rojas-Ponce G, Ntinginya EN, Kalomo M, Minja LT, Kowuor D, Saathoff E, Kroidl A, Heinrich N, Maboko L, Bates M, O’Grady J, Zumla A, Hoelscher M, Rachow A. 2015. Performance of urine lipoarabinomannan assays for paediatric tuberculosis in Tanzania. Eur Respir J 46:761–770. doi:10.1183/09031936.00003315 26113682

[9] Bjerrum S, Schiller I, Dendukuri N, Kohli M, Nathavitharana RR, Zwerling AA, Denkinger CM, Steingart KR, Shah M. 2019. Lateral flow urine lipoarabinomannan assay for detecting active tuberculosis in people living with HIV. Cochrane Database Syst Rev 10:CD011420. doi:10.1002/14651858.CD011420.pub3 PMC6802713 31633805

[10] Herrmann Y, Lainati F, Castro MDM, Mwamba CP, Kumwenda M, Muyoyeta M, Broger T, Heinrich N, Olbrich L, Corbett EL, McMahon SA, Engel N, Denkinger CM. 2022. User perspectives and preferences on a novel TB LAM diagnostic (Fujifilm SILVAMP TB LAM)-a qualitative study in Malawi and Zambia. PLoS Glob Public Health 2:e0000672. doi:10.1371/journal.pgph.0000672 PMC10021253 36962216

[11] Douglas TA, Tamburro D, Fredolini C, Espina BH, Lepene BS, Ilag L, Espina V, Petricoin EF, Liotta LA, Luchini A. 2011. The use of hydrogel microparticles to sequester and concentrate bacterial antigens in a urine test for Lyme disease. Biomaterials 32:1157–1166. doi:10.1016/j.biomaterials.2010.10.004 PMC3019571 21035184

[12] Steinberg HE, Russo P, Angulo N, Ynocente R, Montoya C, Diestra A, Ferradas C, Schiaffino F, Florentini E, Jimenez J, Calderón M, Carruthers VB, Gilman RH, Liotta L, Luchini A. 2018. Toward detection of toxoplasmosis from urine in mice using hydro-gel nanoparticles concentration and parallel reaction monitoring mass spectrometry. Nanomedicine 14:461–469. doi:10.1016/j.nano.2017.11.020 PMC5844831 29203146

[13] Shafagati N, Patanarut A, Luchini A, Lundberg L, Bailey C, Petricoin E 3rd, Liotta L, Narayanan A, Lepene B, Kehn-Hall K. 2014. The use of nanotrap particles for biodefense and emerging infectious disease diagnostics. Pathog Dis 71:164–176. doi:10.1111/2049-632X.12136 PMC7108521 24449537

[14] Paris L, Magni R, Zaidi F, Araujo R, Saini N, Harpole M, Coronel J, Kirwan DE, Steinberg H, Gilman RH, Petricoin EF, Nisini R, Luchini A, Liotta L. 2017. Urine lipoarabinomannan glycan in HIV-negative patients with pulmonary tuberculosis correlates with disease severity. Sci Transl Med 9:eaal2807. doi:10.1126/scitranslmed.aal2807 PMC6037412 29237757

[15] Iqbal NT, Ahmed K, Qamar FN, Shaheen F, Mehnaz A, Arif F, Saeed AA, Yousuf AM, Raza SF, Sultana S, Qureshi SM, Siddiqi SA, Houpt E, Thomas T. 2020. Antibody-secreting cells to diagnose *Mycobacterium tuberculosis* infection in children in Pakistan. mSphere 5:e00632-19. doi:10.1128/mSphere.00632-19 PMC7002306 32024709

[16] Graham SM, Cuevas LE, Jean-Philippe P, Browning R, Casenghi M, Detjen AK, Gnanashanmugam D, Hesseling AC, Kampmann B, Mandalakas A, Marais BJ, Schito M, Spiegel HML, Starke JR, Worrell C, Zar HJ. 2015. Clinical case definitions for classification of intrathoracic tuberculosis in children: an update. Clin Infect Dis 61Suppl 3:S179–87. doi:10.1093/cid/civ581 PMC4583568 26409281

[17] Stop TB Partnership/Global Drug Facility. 2018. Alere determine TB LAM ag assay for detection of Mycobacterium Tuberculosis complex, mentor4TB Toolkit. StopTB. Geneva, Switzerland

[18] 2023. Determine TB LAM Ag: package insert Abbott, Chicago, IL, USA.

[19] World Health Organization. 2024. Target product profile for tuberculosis diagnosis and detection of drug resistance. World Health Organization, Geneva, Switzerland.

[20] World Health Organization. 2019. Lateral flow urine lipoarabinomannan assay (LF-LAM) for the diagnosis of active tuberculosis in people living with hiv: policy update World Health Organization, Geneva, Switzerland.

[21] Branigan D. 2023. Tuberculosis diagnostics pipeline report. Treatment Action Group, New York, NY, USA.

[22] Foysal KH, Seo SE, Kim MJ, Kwon OS, Chong JW. 2019. Analyte quantity detection from lateral flow assay using a smartphone. Sensors (Basel) 19:4812. doi:10.3390/s19214812 PMC6864604 31694281

[23] Bermejo-Peláez D, Medina N, Álamo E, Soto-Debran JC, Bonilla O, Luengo-Oroz M, Rodriguez-Tudela JL, Alastruey-Izquierdo A. 2023. Digital platform for automatic qualitative and quantitative reading of a cryptococcal antigen point-of-care assay leveraging smartphones and artificial intelligence. J Fungi (Basel) 9:217. doi:10.3390/jof9020217 PMC9961444 36836331

[24] Van Hoving DJ, Lahri S, Lategan HJ, Nicol MP, Maartens G, Meintjes G. 2019. Brief report: real-world performance and interobserver agreement of urine lipoarabinomannan in diagnosing HIV-associated tuberculosis in an emergency center. J Acquir Immune Defic Syndr 81:e10–e14. doi:10.1097/QAI.0000000000002002 PMC6522362 30865176

[25] Makumbi S. 2023. Yield and operational challenges of including urine LAM testing in intensified TB case-594 finding among outpatients with HIV: Experience from the TB-SCRIPT trial. The Union World Conference On Lung Health.

[26] Bulterys MA, Wagner B, Redard-Jacot M, Suresh A, Pollock NR, Moreau E, Denkinger CM, Drain PK, Broger T. 2019. Point-Of-care urine lam tests for tuberculosis diagnosis: a status update. J Clin Med 9:111. doi:10.3390/jcm9010111 PMC7020089 31906163

[27] Broger T, Nicol MP, Sigal GB, Gotuzzo E, Zimmer AJ, Surtie S, Caceres-Nakiche T, Mantsoki A, Reipold EI, Székely R, Tsionsky M, van Heerden J, Plisova T, Chikamatsu K, Lowary TL, Pinter A, Mitarai S, Moreau E, Schumacher SG, Denkinger CM. 2020. Diagnostic accuracy of 3 urine lipoarabinomannan tuberculosis assays in HIV-negative outpatients. J Clin Invest 130:5756–5764. doi:10.1172/JCI140461 PMC7598043 32692731

[28] Sigal GB, Pinter A, Lowary TL, Kawasaki M, Li A, Mathew A, Tsionsky M, Zheng RB, Plisova T, Shen K, Katsuragi K, Choudhary A, Honnen WJ, Nahid P, Denkinger CM, Broger T. 2018. A novel sensitive immunoassay targeting the 5-Methylthio-d-Xylofuranose-Lipoarabinomannan epitope meets the WHO’s performance target for tuberculosis diagnosis. J Clin Microbiol 56:e01338-18. doi:10.1128/JCM.01338-18 PMC6258851 30257899

[29] Huerga H, Bastard M, Lubega AV, Akinyi M, Antabak NT, Ohler L, Muyindike W, Taremwa IM, Stewart R, Bossard C, et al.. 2023. Novel FujiLAM assay to detect tuberculosis in HIV-positive ambulatory patients in four African countries: a diagnostic accuracy study. Lancet Glob Health 11:e126–e135. doi:10.1016/S2214-109X(22)00463-6 PMC9747168 36521944

[30] Connelly JT, Grant B, Munsamy V, Pym A, Somoskovi A. 2019. Lipoarabinomannan point-of-care tests: evaluation with fresh samples needed. Lancet Infect Dis 19:1053. doi:10.1016/S1473-3099(19)30475-X 31559954

[31] Broger T, Muyoyeta M, Kerkhoff AD, Denkinger CM, Moreau E. 2020. Tuberculosis test results using fresh versus biobanked urine samples with FujiLAM. Lancet Infect Dis 20:22–23. doi:10.1016/S1473-3099(19)30684-X 31876492

[32] Nel JS, Lippincott CK, Berhanu R, Spencer DC, Sanne IM, Ive P. 2017. Does Disseminated nontuberculous mycobacterial disease cause false-positive determine TB-LAM lateral flow assay results? a retrospective review. Clin Infect Dis 65:1226–1228. doi:10.1093/cid/cix513 28575238

