# Supplementary material for: Nanoparticle capture of urinary lipoarabinomannan for diagnosing childhood tuberculosis
Source: J Clin Microbiol. 2026 Oct 12;64(11):e01559-25. doi: 10.1128/jcm.01559-25 (PMC13753829; doi:10.1128/jcm.01559-25)
Supplement: Supplementary file 1 [file jcm.01559-25-s0001.docx]

**Supplemental Material**

**Appendix A. Inclusion and exclusion criteria**

| Inclusion criteria |  | Exclusion Criteria |
| --- | --- | --- |
| Consent of parent or legal guardian and assent (if applicable) of participant |  | Currently receiving TB therapy |
| Age >1 years, ≤ 18 years |  | Received BCG vaccine or TST in the past 8 weeks |
| Resident of Karachi, Pakistan |  | Immunocompromised/immunosuppressed status including HIV |
| Case Participants only |  | **Control Participants only** |
| - Two or more symptoms of pulmonary or extrapulmonary TB - Initiated therapy for TB |  | - Known TB exposure within the past 2 weeks - History of receipt of TB therapy - Severe acute malnutrition |

Confirmed TB

Microbiologic confirmation of TB (positive Gene Xpert or Mtb culture) from a clinical specimen

At least 1 symptom suggestive of TB

Unconfirmed TB

Bacteriological confirmation of TB not obtained

At least 2 of the following:

- Cough > 2 weeks, weight loss, fever > 1 week, lethargy
- Chest xray consistent with TB
- Known exposure to TB or immunologic evidence of TB infection
- Positive clinical response to TB treatment

**Appendix B. Case definitions**
